# Seamlessly integrated textile electrodes and conductive routing in a garment for electrostimulation: design, manufacturing and evaluation

**DOI:** 10.1038/s41598-023-44622-5

**Published:** 2023-10-13

**Authors:** Emanuel Gunnarsson, Kristian Rödby, Fernando Seoane

**Affiliations:** 1 Textile Materials Technology, Department of Textile Technology, Faculty of Textiles, Engineering and Business Swedish School of Textiles, University of Borås, Borås, Sweden; 2 Institute for Clinical Science, Intervention and Technology, Karolinska Institutet, Stockholm, Sweden; 3 Department of Medical Care Technology, Karolinska University Hospital, 14157 Huddinge, Sweden; 4 Department of Clinical Physiology, Karolinska University Hospital, 14157 Huddinge, Sweden

**Keywords:** Engineering, Electrical and electronic engineering

## Abstract

Electro-stimulation to alleviate spasticity, pain and to increase mobility has been used successfully for years. Usually, gelled electrodes are used for this. In a garment intended for repeated use such electrodes must be replaced. The Mollii-suit by the company Inerventions utilises dry conductive rubber electrodes. The electrodes work satisfactory, but the garment is cumbersome to fit on the body. In this paper we show that knitted dry electrodes can be used instead. The knitted electrodes present a lower friction against the skin and a garment is easily fitted to the body. The fabric is stretchable and provides a tight fit to the body ensuring electrical contact. We present three candidate textrodes and show how we choose the one with most favourable features for producing the garment. We validate the performance of the garment by measuring three electrical parameters: rise time (10–90%) of the applied voltage, net injected charge and the low frequency value of the skin–electrode impedance. It is concluded that the use of flat knitting intarsia technique can produce a garment with seamlessly integrated conductive leads and electrodes and that this garment delivers energy to the body as targeted and is beneficial from manufacturing and comfort perspectives.

## Introduction

Wearable electronic systems will be of great importance in the future healthcare due to its potential to lower the cost of surveillance and at the same time offering better monitoring capabilities. These kinds of systems will be based on garments that people, including elderly, are familiar with such as t-shirts, pants or a vest (i.e. undershirt or “tank top”). The electrical systems will be integrated into the garments with varying degrees of integration. This will make home monitoring easily administrated since the patients can put the monitoring devices on by themselves or with the aid of a home care worker. In order to be easily applied such smart textile systems needs to rely on the use of dry electrodes that has no need of preparation before usage.

Wearable electronic systems for both sensing and/or stimulating purposes can benefit from the use of dry electrodes as an interface between the body and the electronic device(s) in several aspects, such as ease of use and administration and chemical stability over time^1^. This interface presents some certain contact impedance to the system, as does conventional electrodes (e.g. gelled Ag/AgCl), henceforth the term “contact impedance” will imply the skin–electrode impedance. The magnitude and characteristics of the contact impedance depends on a multitude of factors, some of them are factors concerning the subject, e.g. age, body composition, moist, gender, others are factors that stems from the electrode itself, e.g. area, material properties, surface topology, contacting pressure. Previous studies about both stimulation and detection of bio-potentials have shown that a stable and high enough contacting pressure is of importance in order to keep the contact impedance stable^1–3^. If the contact impedance is varying during monitoring the signal fidelity might be severely affected and in the case of stimulation, there is a risk of current density constriction.

The dry electrodes used today are often made of a conductive rubber or silicone material^1,4^. These rubber materials present a rather high friction against the human skin which in a sense is good since it promotes a stable positioning of the electrode once applied. On the other hand, when these electrodes are integrated into a garment that needs to have a tight fit so as to keep an as stable as possible contact impedance, their high friction constitutes an obstacle for getting the garment in place. This will be especially pronounced on patients with significant sensibility as is often the case with persons having cognitive and/or motoric dysfunction.

The idea for the study presented in this paper originates from the aforementioned shortcomings. The Mollii-suit is a Swedish medical device that is intended for aiding patients with different neurological conditions^5^. It consists of an over-all with 58 conductive rubber electrodes placed on different locations. The electrodes are connected to an electrostimulation device worn at the waist of the wearer. The conductive paths between the electrodes and the electro-stimulation device consist of canals where conductive elastic bands reside^6^. In 2018 a project was set up to investigate if a garment could be manufactured using intarsia knitting for producing both conductive leads and electrodes in a single manufacturing step^7^. Since the effect of electrostimulation for suppressing tremor was gaining interest in the medical research^8^, the company had a wish to try their device on patient with Parkinson’s disease and wanted a garment that would be easier to put on the patients. They had testimonies from their users that the conductive rubber electrodes posted an obstacle when putting the garment on and discomfort during the wearing of it originating certain discomfort^9^. It should be noted that the electro-therapy of the Mollii-suit is applied to promote reciprocal inhibition. The treatment is usually applied as the wearer is sitting still or lying still^10,11^. The therapy is not in the first hand intended to be applied while the wearer is moving.

One way of alleviating this problem would be to use textile electrodes made of conductive yarns that possess the qualities usually associated with textiles, i.e. low friction against the skin, drapability and high comfort. If these electrodes together with the conductive pathways, necessary for making the connection between any measuring or stimulating device and the electrodes themselves, could also be made in a single textile manufacturing process the production cost of the whole system would be decreased.

There has been reports on previous investigations into such garments, and even before the birth of the notion of “smart textiles”, fabrics were utilised as an intermediate layer between metallic electrodes and the skin. Since the demarcation of smart textiles around the turn of the century and onwards there has been investigations into this subject with an increase in reported work during the last decade.^2,12–19^. In a recent review of textile electrodes for stimulation Euler et al. has mapped out much of the work done hitherto. They found that the samples investigated ranged over most common textile techniques: printing, coating, knitting, weaving and non-woven methods^20^. In a related paper by the same group, they found that a high density of yarns if favourable, and that in the case of dry electrodes that an uneven surface is beneficial^21^. There are virtually endless ways to vary the construction of a knitted textrode and we chose, based on our prior experience and on the information found in the literature, two constructions where the main difference was the “unevenness” of the surface, types C and D. In addition to those we also investigated sample type B which has an even more even surface. Asperities and patterns are on a length scale of approximately 0.4 mm for type B, 0.83mm for type D and 3.3mm for type C. In the aforementioned review, and also from the searches we have done for this work, it seems that only one paper uses flat-knitting intarsia technique^17^ and possibly, though not explicitly stated, one paper uses circular knitting intarsia^2^. As far as we have seen, none of the reported work has utilised intarsia to produce both the conductive pathways and the electrodes in one single production step.

In this paper we present the design and manufacturing of first a set of candidate textile electrodes and second a garment. The garment was manufactured with only two production steps: a single knitting step resulting in fabrics with electrodes, conductive pathways and connection-points for an intended electrostimulation-device, and a single confection step sewing the fabrics together to form a pair of pants. No finishing was done on the samples. We also present the results of in-vivo measurements of the waveforms during TENS stimulation and the skin–electrode impedance of textile electrodes made with different bindings and compare them with conventional dry electrodes.

## Method

Firstly, a set of separate textile electrodes were produced using different textile materials and binding techniques. The textile electrodes have been characterized and the electrostimulation function has been studied. For the later a comparison with commercially used electrodes: gelled electrodes and dry rubber electrodes, has been included. The dry rubber electrodes were considered the benchmark since they are currently used in Mollii-suit^6^. After the above initial steps were undertaken, an evaluation of which of the candidate electrodes performed best was undertaken and a garment (a pair of pants) was produced by first producing a fabric that contained both electrodes (the binding chosen in the aforementioned evaluation) and leads (conductive pathways) in a single knitting step and one confection step to complete the garment. This garment was then subjected to the same kind of testing as the separate electrodes to validate the functionality. This study was conducted following ethical approval DNR 274–11 from the regional ethics committee of Gothenburg and was conducted in accordance with the declaration of Helsinki. Informed consent was obtained from the participant.

### Materials

#### Electrode material

**Figure 1:**
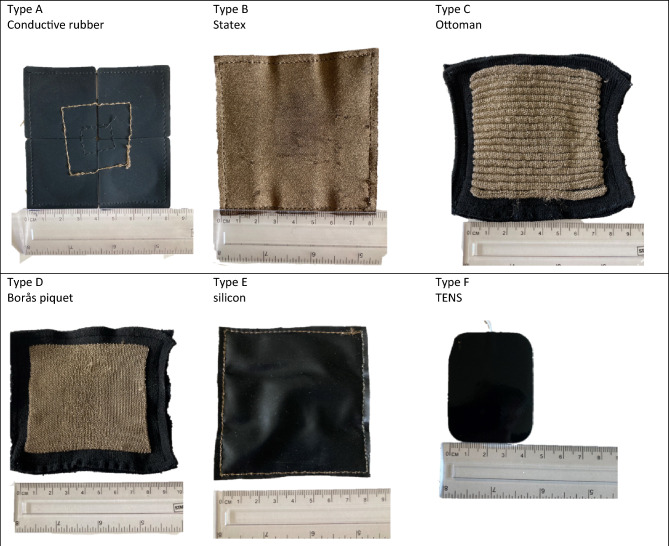
Photographs of the six types of electrodes under study. Type A is the benchmark electrode used as a reference. Types B and E are made of commercially available fabrics: Type B is silverised, Type E has a conductive silicon coating. Types C and D are knitted in house. Type F is a commercial gelled TENS electrode that comes with the TENS machine.

Four types of electrodes were compared: the benchmark was the conductive rubber electrode (type A), this material is currently being used in the Mollii-suit^4^; the next type was a readymade warp-knitted fabric called Shieldex Silitex P130 made out of 78% polyamide and 22% elastomer, the fabric is silver-coated on one side and coated with a conductive silicone on the other side (type B)^22^; the two types made in house both utilises the same kind of yarn: a silver-coated polyamide multifilament with a yarn number of 117/1 2-ply^23^ and the supporting “substrate” for these were done with a multifilament polyester yarn with yarn number 78 dtex 72 filaments plated with a polyamide/lycra 78/78 dtex (types C and D). As the benchmark electrode is made from conductive rubber we also chose to make samples of conductive silicon coating (type E) and to get an indication of how these five types of electrode function compared to ordinary gelled TENS electrodes (type F) such were also tested. It must be noted that the surface area of the TENS electrodes is not equal to the other electrodes, and that sample types E and F are only used for getting indicative numbers, they were never under consideration for implementation in the garment. Photographs of the samples can be seen in Fig. 1. The three sample types B, C and D represent different degrees of macroscopical surface unevenness, where type B is the smoothest, type D is intermediate and type C is the roughest.

#### Electrode manufacturing

**Figure 2:**
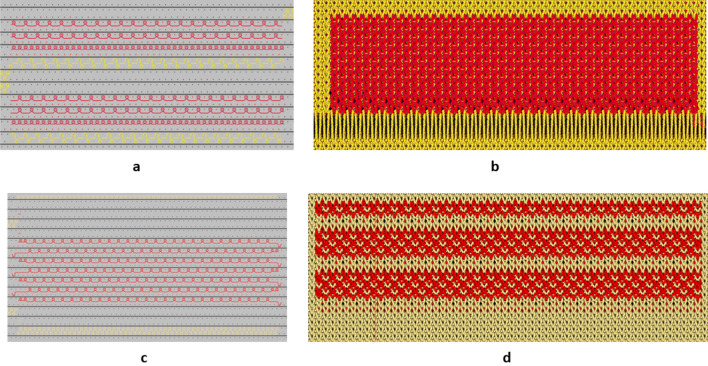
(**a**) Technical view of binding type C. (**b**) simulation view of the same binding. (**c**) Technical view of the binding of type D. (**d**) simulation of the binding of type D. The red yarn is the conductive and the yellow is the substrate yarn.

As an initial test, electrodes were manufactured as patches with Velcro on their backside, one of the Velcro pieces (situated in the middle) was conductive. We set the size of the candidate electrodes to be 64 cm2, and the size of the rubber electrodes was 16 cm^2^ hence, for type A, we stitched 4 of the rubber electrodes together. For them to act as an equipotential surface we used conductive thread for this stitching. For types B and E, we cut pieces of the size 8 × 8 cm out of the warp-knitted fabric and stitched it onto the same kind of backing as for type A. The two types knitted inhouse were: an ottoman pattern (type C) and a variation of a piquet binding, that we label “the Borås piquet”, (type D) were both knitted on a Stoll ADF530-KI 7.2 multi-gauge with 14-gauge needle-bed, using 12 gauge needle. These two types already came with a workable backing out of the knitting machine, hence the Velcro could be stitched directly on the backside of these types. The binding of type C is done by using a full rib binding with 16 courses of single interlock using only the conductive yarn creating the “ripple” and in between the ripples a dual yarn full rib, similar to a birdseye effect, functioning as the binding points to the substrate. Type D is done by first doing a ½-gauge tuck-binding using the substrate yarn, then one course of plain knit with the conductive yarn followed by two courses of ½-gauge plain knit also with the conductive yarn, this is repeated using the previously unused needles. The three textrode types, B, C and D, represent surfaces with “irregularities” on different length scales. For type B both courses and wales are approximately 24/cm, for type C and D there are 10 courses/ cm and 12 wales/cm. The types B and D are both flat on a macroscopic scale whereas type C has its “waves”, thus in effect the irregularities of the different types are on length scales 0.41 mm, 0.83 mm and 3.3 mm respectively. The technical drawings of type C and D can be seen in Fig. 2.

#### Garment fixture for initial in-vivo characterisation

**Figure 3:**
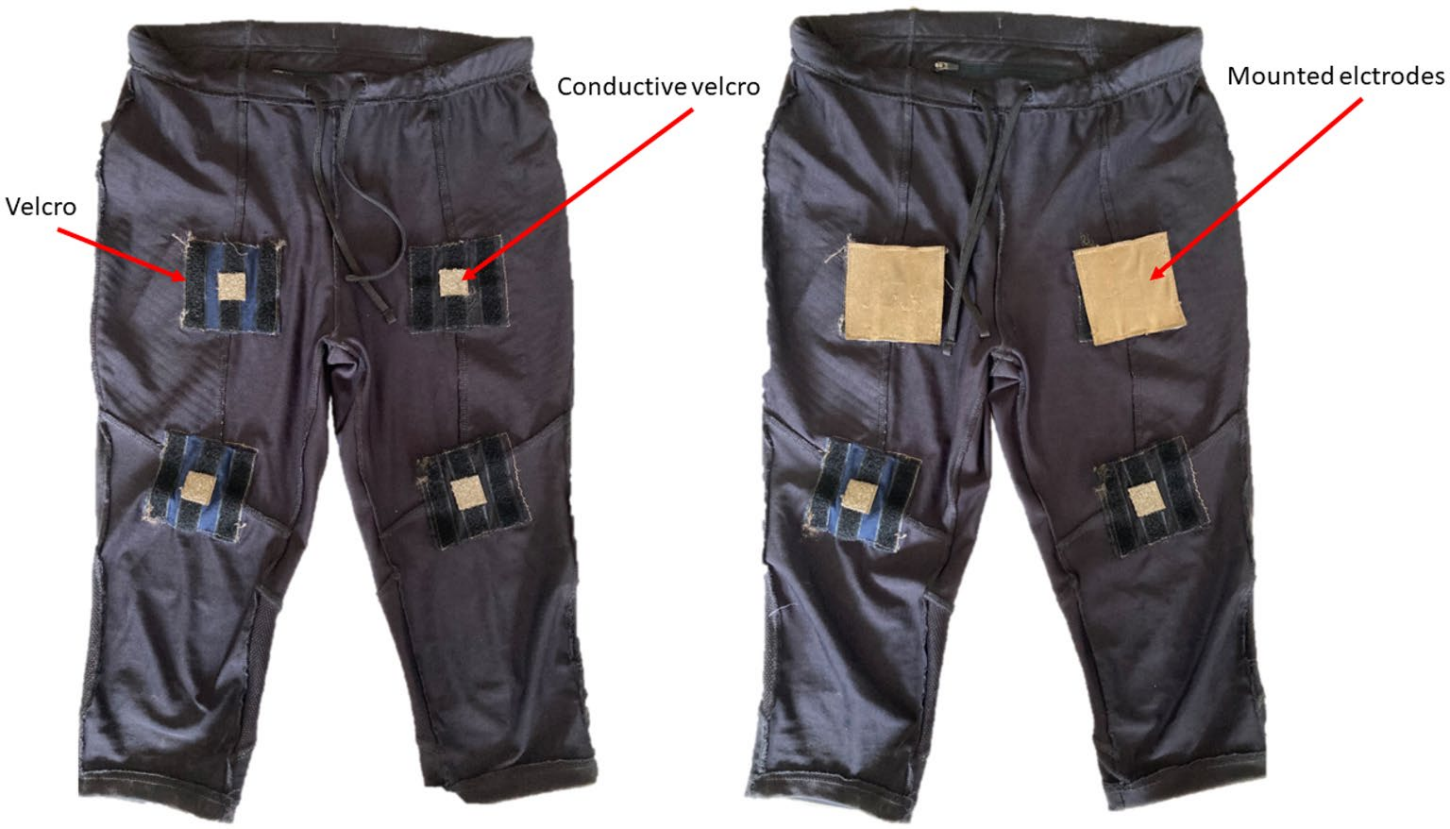
Training pants inside out for fixture of the patch electrodes. The left photo shows the four sites with Velcro, the brighter small squares in the middle is the conductive Velcro connecting the electrode to the outside of the pants. In the right photo two electrodes of type B are mounted for illustration.

For the initial tests, a pair of workout pants were fitted with connections for the patches described in the previous section by stitching conductive Velcro at specific positions. The reason for doing this was to have a mechanical pressure between the skin and the electrode that stayed approximately the same between measurements. These pants can be seen in Fig. 3.

#### Garment manufacturing

**Figure 4:**
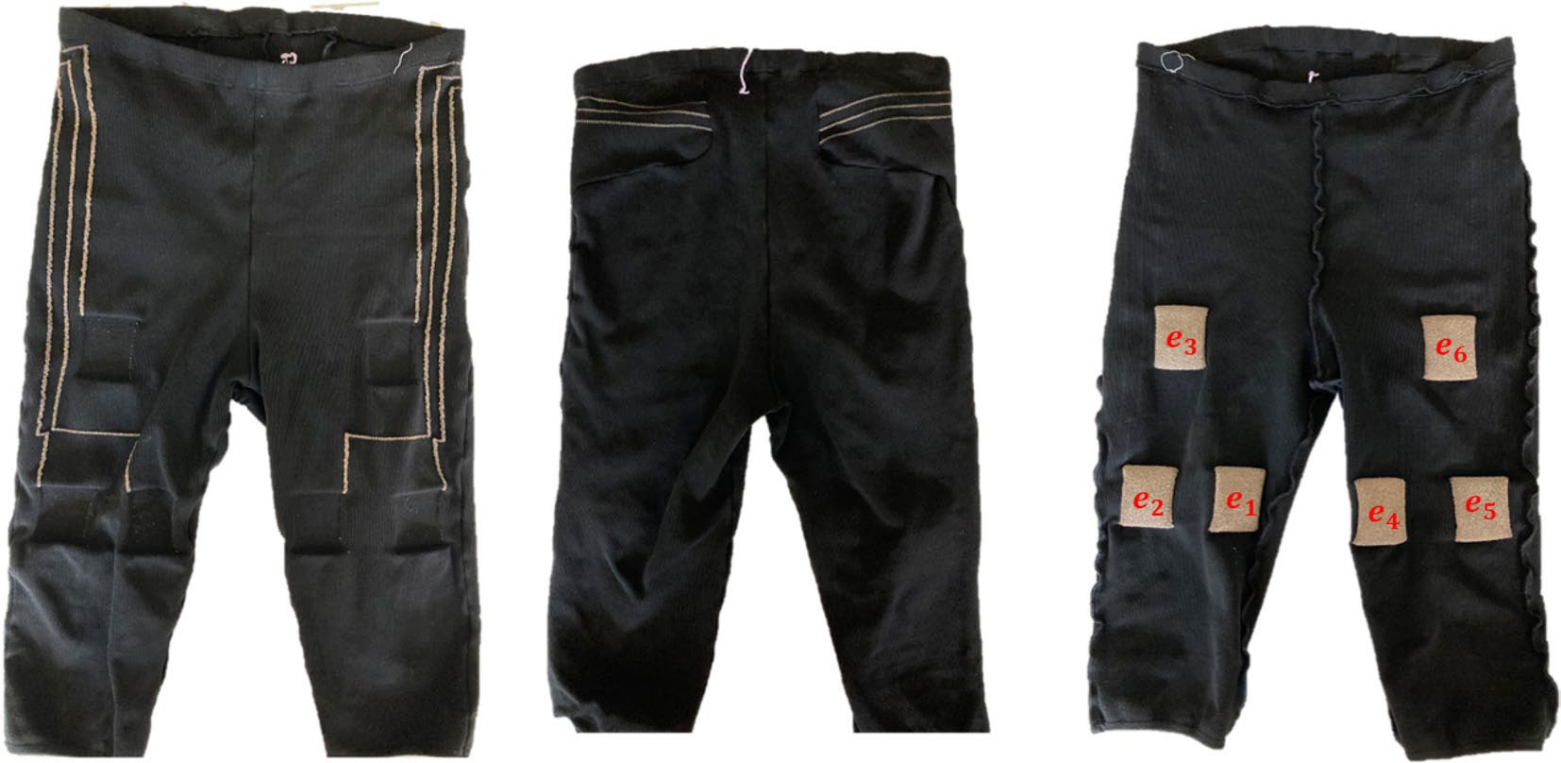
Photos of the pants. Leftmost picture shows the front outside, middle shows the rear outside and rightmost shows the front inside. In the rightmost photo the labelling of the electrodes is indicated.

Once the initial tests were done, we proceeded by manufacturing pieces of fabric that contained both electrodes, conductive pathways and connection-points to a future electrostimulation-device. These pieces were then sewn together to form a pair of pants. The fabric for the pants were knitted with the same yarns as described for electrode types C and D in "Electrode material" section: i.e. a full rib stitching using a multifilament polyester yarn with yarn number 78 dtex 72 filaments plated with a polyamide/lycra 78/78 dtex. The knitting was done on the same machine as described in "Electrode manufacturing" section. The conductive pathways between the connector of a future electrostimulation-device and the electrodes were done by using plain knit for both the silver-coated yarn and the polyester/polyamide/lycra while switching the yarns from one needle bed to the other so as to have the electrodes on the side of the fabric facing the human body and the conductive pathways on the opposite side. Each pair of pants contained six electrodes, three on each leg. The electrodes are labelled $e_{1} ,e_{2} ,e_{3} ,e_{4} ,e_{5} ,e_{6}$. A schematic sketch of the pants can be seen in Fig. 4.

#### Electrical measurement equipment

For characterization measurements in both time domain and frequency domain a digital USB oscilloscope was used. The oscilloscope was a Picoscope 5442D MSO from Picho Technology with flexible resolution with a built-in function generator. The sampling rate while using 3 analogue channels was set to 125 MS/s and the hardware resolution was 15 bits^24^.

To perform the time domain measurements, we used a TENS device from e-caretalk, model Tiq The manufacturer does not specify whether it is delivering a constant current or a constant voltage. What is being stated is the following:

- Pulse amplitude: 0–25 V (200 Hz @ 300, µs) or 0–120 V (2 Hz @ 30 µs), peak into 500 Ω load of each channel
- Pulse rate: 2–200 Hz, adjustable
- Pulse width: 30–300 µs, adjustable
- Wave form: Single-phasic square pulse
- Modes: Burst, modulation and conventional

**Figure 5:**
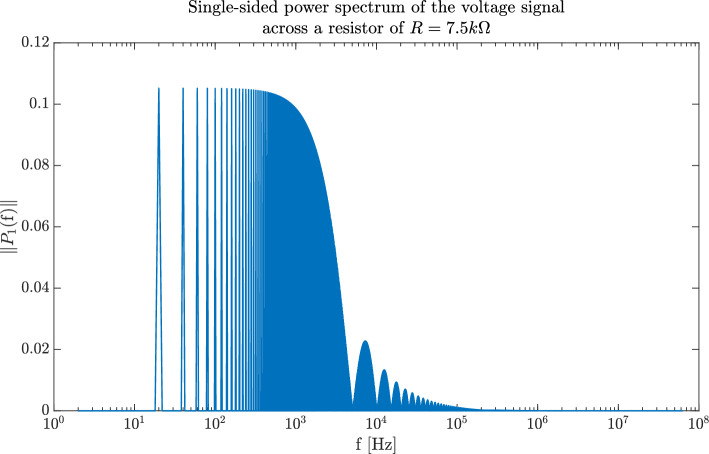
Single-sided power-spectrum of the signal used for the time-domain measurements as taken across a resistor of 7.5 kΩ. Most of the power resides in the frequency span 20 Hz ≤ f ≤ 10 kHz.

A pre-test conducted with the TENS device revealed that it functions as a voltage-controlled source. The program used for the time domain measurements is called P-02, which delivers a monophasic square pulse of 30 µs duration at 20 Hz frequency ($0.6\;\textperthousand$ duty cycle). The energy content of such a signal is mostly situated between the zeroth harmonic (20 Hz) and ca 10 kHz. The single-sided power spectrum of such a signal can be seen in Fig. 5. Hence measurements in the range $1\; {\text{Hz}} \le f \le 50 \;{\text{kHz}}$ will provide the relevant information.

### Electrical characterisation and performance comparison

In order to investigate the behaviour of the electrodes, two types of measurements were done on the samples: first a time domain analysis of the waveform through the whole system consisting of skin–electrode impedance, body impedance and another skin–electrode impedance to verify that energy was being transferred to the body; and second, a frequency response analysis to determine the contact impedance between the candidate samples and the human skin. The time domain measurement, while done using commercial gelled electrodes and the benchmark electrodes (type A), also provided us with the information on where in the frequency domain the energy was situated, hence suggesting the proper frequency span to conduct the FRA measurements in.

#### Time domain characterisation

The intended use of the garment is electro-stimulation of the body, hence a TENS device was used for testing. The operation of the TENS device was not clearly stated in the documentation of the device which led us to first investigate whether the device was designed to deliver a constant current or a constant voltage to the body. To investigate this, we connected the device to a series of loads in the form of ordinary resistors ranging from $100 \;\Omega \le {\text{R}} \le 5100 \;\Omega .$ After this was done, the time domain recordings of the garment were done by connecting the TENS device between two electrodes while the garment was worn by a healthy male test subject. The USB oscilloscope was used for recording the signals. Two signals were recoded: Channel A recorded the voltage across the whole load consisting of two contact impedances, the body impedance and a reference resistor, all in series; while Channel B recorded the voltage across the reference resistor. Since the value of the reference resistor was well known to us (${R}_{shunt}=100\;\Omega$), the latter measurement provided us with a means to determine the current through the system. Formally this can be expressed as

1 $$\begin{array}{c}{V}_{A}=I \left({Z}_{c1}+{Z}_{b2}+{Z}_{b3}+{Z}_{c2}+{R}_{shunt}\right)\\ {V}_{B}=I {R}_{shunt}\\ V={V}_{A}-{V}_{B}\approx {V}_{A}\end{array}$$

where the last approximation is justified by the large difference in magnitude between ${R}_{shunt}$ and the other impedances.

**Figure 6:**
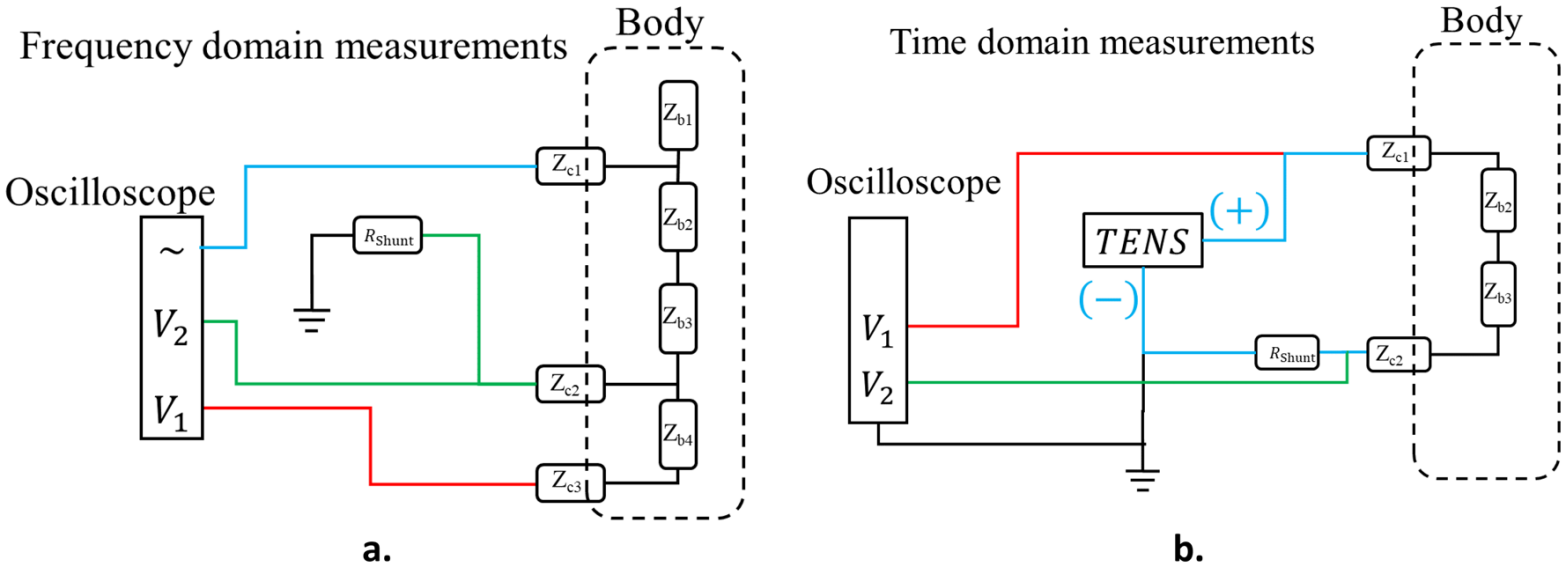
Schematic overview of the measurements. A: the frequency domain measurement setup. The contact Z_c2 is the one that is being characterized. B: the time domain measurement setup. The load in this case includes two contacts and the body impedance.

A schematic view of the measurement setup can be seen in Fig. 6b. Two parameters from the time domain measurements were chosen for the characterisation: the rise time of the voltage signal, defined as the time it takes the voltage to go from 10% of the maximum value to 90% of the maximum value;

2 $${t}_{10-90 }\equiv {\left.t\right|}_{V=0.9{V}_{\mathrm{max}}}- {\left.t\right|}_{V=0.1{V}_{\mathrm{max}}}$$

and the total net injected charge, defined as the integral of the current signal w.r.t. time.

3 $$q= \underset{0}{\overset{T}{\int }}i\left(t\right)dt$$

where $T$ is the period of the pulse.

Furthermore, these measurements also gave us a picture of in what frequency range that would be relevant to look at the impedance.

#### Frequency response analysis

The skin–electrode impedance measurements were conducted by three-point in-vivo measurements. A detailed description of the method is published in another paper by us^25^. The instrument used for the measurements was the same USB oscilloscope in conjunction with a third-party software called FRA4picoscope^26^. The measurement principle is depicted in the left picture of Fig. 6a. The assumption is that no current is running towards the terminal labelled ${V}_{a}$ and hence the value of ${Z}_{b}$ (which is the DUT) can be extracted as described below. The signal obtained from the software consisted in the logarithmic gain of the DUT together with the phase angle, i.e.

4 $$G\left(f\right)=20{\mathrm{log}}_{10}\left(\left|\frac{{V}_{b}}{{V}_{a}}\right|\right)$$

and

5 $$\varphi \left(f\right)=\mathrm{atan}\left(\frac{{V}_{b}}{{V}_{a}}\right)$$

In order to retrieve the impedance of ${Z}_{b}$ we note that

$$G\left(f\right)=20{\mathrm{log}}_{10}\left(\frac{I{R}_{s}}{I\left({Z}_{b}+ {R}_{s}\right)}\right)\Rightarrow$$

6 $$\Rightarrow Z_{b} \left( f \right) = R_{s} \left( {10^{{ - \left( {{\raise0.7ex\hbox{$G$} \!\mathord{\left/ {\vphantom {G {20}}}\right.\kern-0pt} \!\lower0.7ex\hbox{${20}$}}} \right)}} - 1} \right)$$

First, we measured all four types of electrodes by letting a test-person wear the workout pants after mounting them with one type of electrode at all four positions. The gain was then recorded in the frequency range of $1 \;\text{Hz}\le f\le 50 \;\text{kHz}$. This measurement was then repeated using the knitted garment. The spectrograms are fitted to the model:

7 $$Z=\frac{{R}_{p}}{1+{R}_{p}Q {\left(j \omega \right)}^{\alpha }}+{R}_{s}$$

As the frequency tends towards zero this model’s magnitude tends towards ${\left|Z\right|}_{\omega \to 0}\to {R}_{p}+{R}_{s}$.

### Friction test

To confirm our initial expectations that the textrodes present a lower friction against the skin an indicative test of the friction was conducted. The test consisted of two measurements: first against a smooth board and second against the human skin of the anterior side of the thigh (approximately from the Iliacus and Psoas major to the inferior attachment of Rectus femoris). The samples were attached to a bag with a deadweight and slid across the underlying surface. The static and kinetic coefficients of friction are taken as the peak value and the mean value of the latter part of the displacement respectively.

## Results

**Figure 7:**
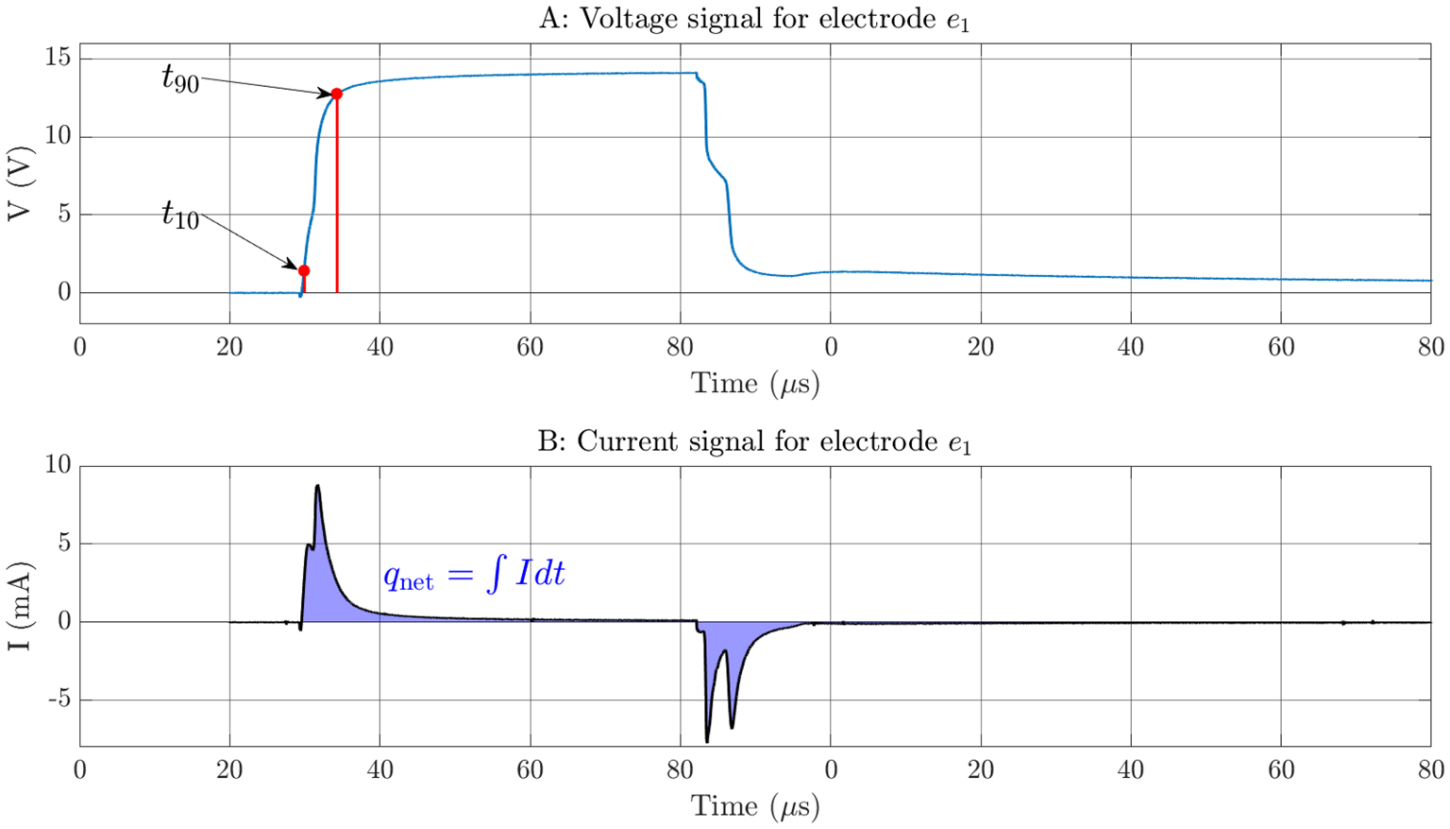
In the upper pane the voltage waveform is depicted. The times at which the voltage reaches 10% and 90% of the maximum value are indicated as ${t}_{10}$ and ${t}_{90}$ respectively. In the lower pane the current waveform is depicted. The shaded area under the graph indicates the integral of the signal w.r.t. time.

**Figure 8:**
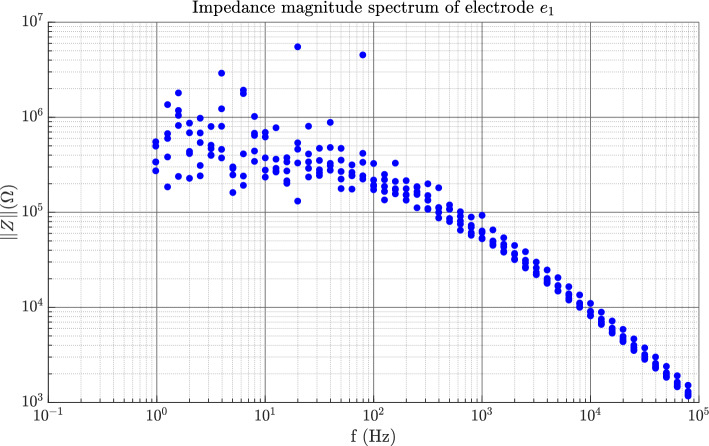
An example of the impedance spectrogram for electrode e_1 of the garments. For frequencies lower than ca 100 Hz there is a large variability and at higher frequencies all five measurements tend towards the same line.

**Figure 9:**
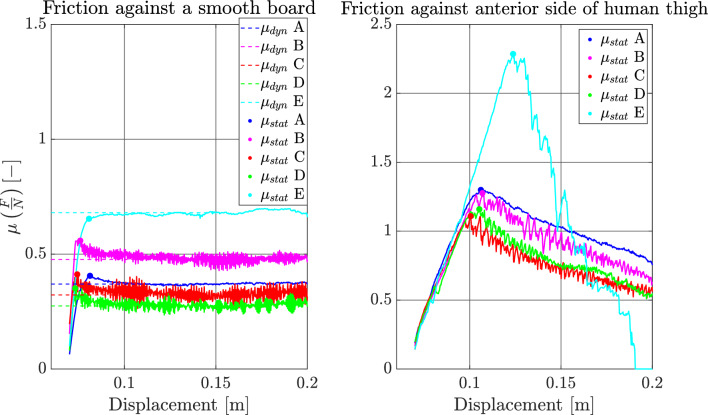
Results of the friction tests. In the test against a smooth board both static and dynamic coefficients are easily identified. In the test against the skin there is no flat region for any of the samples. The tendency in the skin test is that that the more uneven and softer surfaces of the textrodes present a lower friction compared to the rubber and silicon samples.

In the time domain measurements, the results reflect the behaviour of the whole system, i.e. two skin–electrode interfaces and the tissue between these contacts and also the internal impedance of the TENS-device. Due to the complexity of the system the waveshapes are rather complicated and contain many details. We have chosen two parameters that are crucial for evaluating the performance of the electrodes, given the electro stimulation application, namely the rise time of the voltage and the net injected charge. An example of how the time domain measurement curves look can be seen in Fig. 7, here for electrode ${e}_{1}$ of the garments. The upper pane shows the voltage signal that is used for measuring the rise time, and the lower pane shows the current signal that is used to calculate the total charge injected into the system. The integral gives a measure of the net injected amount of charge to the system. For the frequency domain measurements, we have chosen one parameter: the magnitude as the frequency tends toward zero. In Fig. 8 the five impedance spectrograms for electrode ${e}_{1}$ are displayed. All measurements follow the same tendency, there is a rather flat response between 1 Hz and slightly less than 100 Hz whereafter a roll-off commences. As can be seen the variability is rather large towards the lower frequencies but as the frequency increases all measurements tend to fall into the same line. The results of the friction test can be seen in Fig. 9. In the friction against the board the static and dynamic coefficients are easily identified. In the friction against the skin a peak can still be identified for the static coefficient but there is no steady part to identify the dynamic coefficient.

### Results for patches

**Figure 10:**
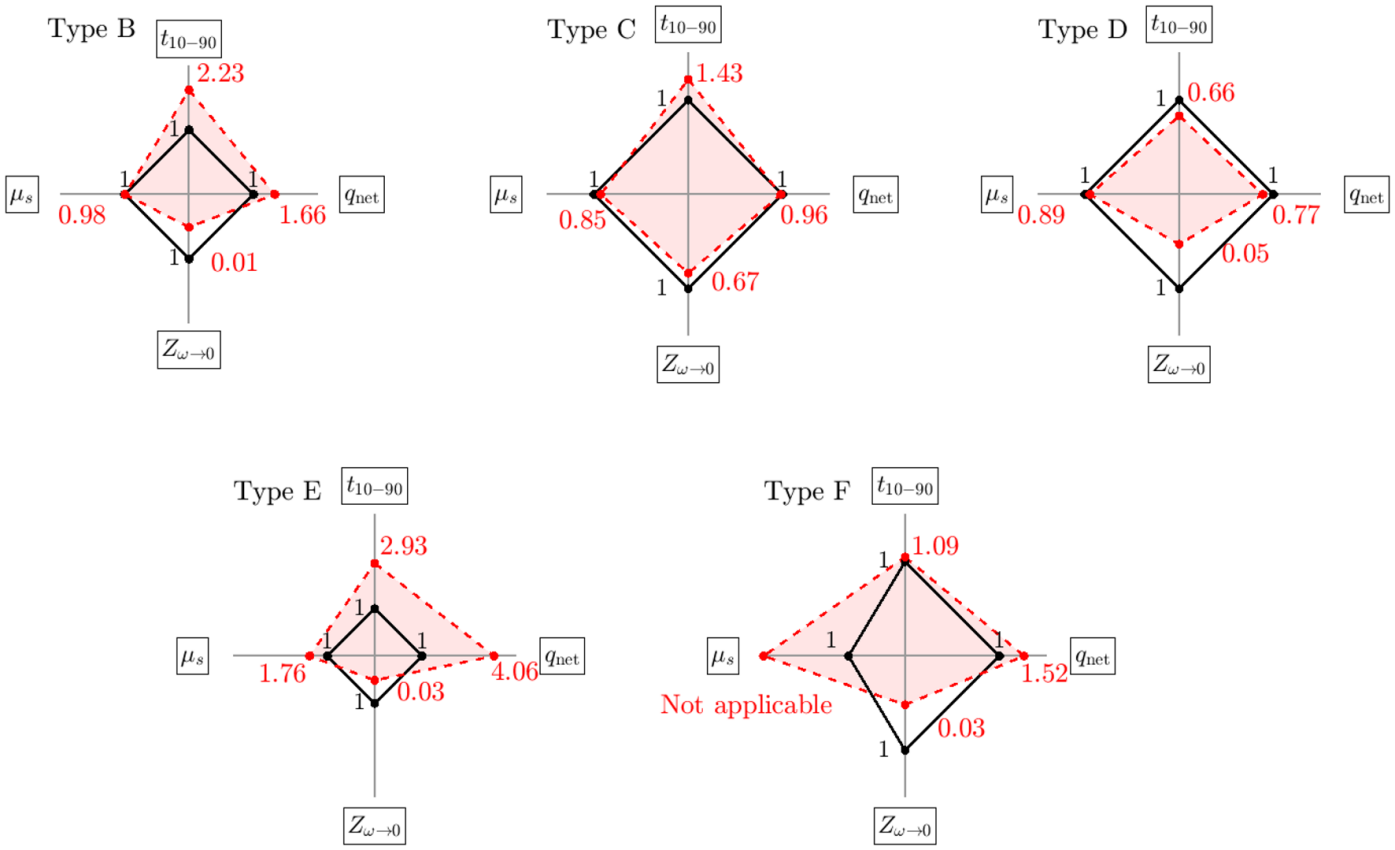
Spider charts for the patches. The four axes are the rise-time, the net injected charge, the low frequency value of the impedance and the static coefficient of friction as measured on the human skin. The solid line black lines represent the benchmark electrode (Type A) with vertices at (1,1,1,1).

The measurement setup for the time domain analysis does not allow us to measure the current through individual contacts. It is thus the current through the whole system we measure. This is in contrasts to the frequency domain analysis where the impedance of individual contacts are measured. The impedance spectrograms also display the typical behaviour of the kind of networks aforementioned. Hence to get a clearer overview of the results, we aggregate four parameters chosen for the characterisation, namely: the rise-time of the voltage pulse, the net injected charge, the magnitude of the impedance as the frequency tends toward zero and the peak value of the friction against the skin. These aggregated results are displayed as four axes spider charts in Fig. 10. In the spider charts the values are normalised to the benchmark (Type A). For transparency all the parameter values before normalisation are collected in Table 1 where the columns are: electrode type, the risetime, the net charge, the magnitude of the impedance between $1\le f\le 40\, \mathrm{Hz}$ and the peak value of the friction against the skin. For the time domain values the mean values are taken over 63 pulses. For the frequency domain the mean is taken over five measurements. For the friction test the mean is also taken over five measurements. So, the normalisation is done as

**Table 1:** The four chosen parameters for evaluation of the candidate electrode types.

Electrode	Risetime $\widehat{\tau }\pm \sigma \left[\mu s\right]$	Net charge $\widehat{q}\pm \sigma$ injected $\left[nC\right]$	Impedance ${\left.\left|Z\right|\right|}_{\omega \to 0}\left[k\Omega \right]$	Static coefficient o friction ${\mu }_{s}\left[-\right]$
A	$4.98\pm 0.62$	$110.67\pm 31.00$	$1 500.0$	1.30
B	$11.10\pm 0.22$	$183.59\pm 2.57$	$20.0$	1.28
C	$7.14\pm 0.5$	$106.53\pm 7.73$	$1 000.0$	1.11
D	$3.30\pm 1.01$	$85.11\pm 1.54$	$81.1$	1.16
E	$14.57\pm 0.25$	$449.71\pm 1.45$	$45.0$	2.29
F	$5.45\pm 0.51$	$167.95\pm 5.23$	$49.3$	Not applicable

$${t}_{10-90}=\frac{{t}_{10-90}\left(i\right)}{{t}_{10-90 }\left(A\right)}$$

$${q}_{net}=\frac{{q}_{net}\left(i\right)}{{q}_{net}\left(A\right)}$$

8 $${Z}_{\omega \to 0}=\frac{{Z}_{\omega \to 0}(i)}{\begin{array}{c}{Z}_{\omega \to 0}\left(A\right)\\ {\mu }_{s}=\frac{{\mu }_{s}(i)}{{\mu }_{s}(A)}\end{array}}$$

For instance, for Type B the values are:

$${t}_{10-90}=\frac{11.10}{4.98}\approx 2.23, {q}_{net}=\frac{183.59}{110.67}\approx 1.66, {Z}_{\omega \to 0}=\frac{20k\Omega }{1.5M\Omega }\approx 0.01 {\mu }_{s}=\frac{1.27}{1.30}\approx 0.98$$

A value of 1 on any axis suggests equivalence with the benchmark, a value lower than 1 on the rise time, impedance and friction axes suggests improvement and a value greater than 1 on the net charge axes also suggests an improvement.

### Results for pants

**Figure 11:**
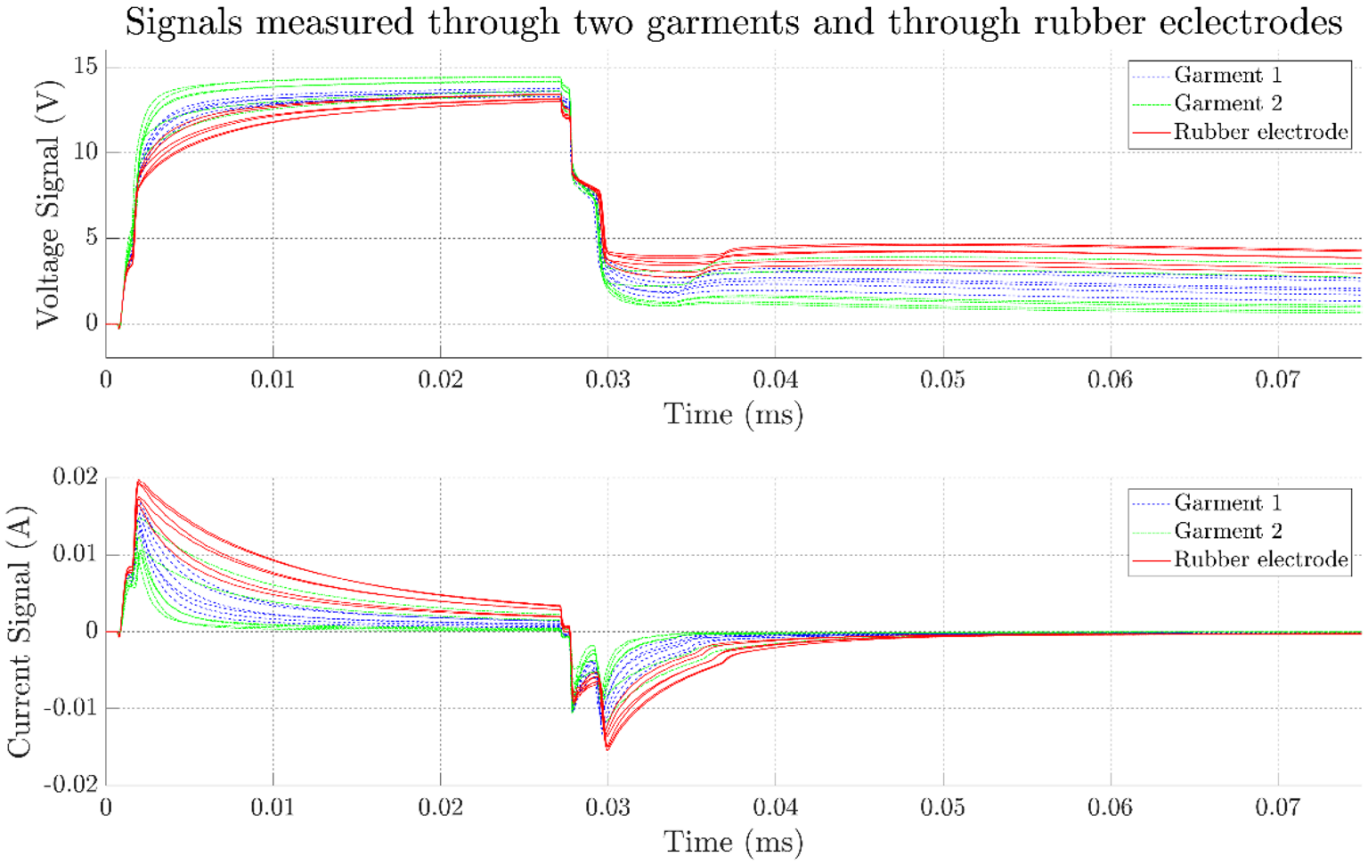
Voltage and current signals for two garments and rubber electrodes. The six lines for each type correspond to the individual electrodes. The curves are the mean of 63 pulses each.

In Fig. 11 the voltage and current signals for two garments can be seen, as an indication also the signals of the commercial rubber electrodes are included in the figure. From the curves it is evident that both garments have lower rise-times than the benchmark (solid red line). Garment 2(dash dotted green line) exhibit the lowest risetimes and the highest variation between the electrodes while garment 1 (dashed blue line) has risetimes between garment 2 and the benchmark for all electrodes. The lower pane shows the corresponding current signals, the initial peak value falls of slowest for the benchmark and fastest for Garment 2 with the values of Garment 1 at rates intermediate between the latter two. The risetimes and net injected charges for the garments and the rubber electrodes are tabulated in Table 2. The risetimes for both garments are generally smaller compared to the rubber electrodes. The net injected charges are also generally smaller.

**Table 2:** The risetimes and net injected charge for two garments and rubber electrodes.

	Risetime $\widehat{{\varvec{\tau}}}\pm{\varvec{\sigma}}\left[{\varvec{\mu}}{\varvec{s}}\right]$			Net charge $\widehat{{\varvec{q}}}\pm{\varvec{\sigma}}\left[{\varvec{n}}{\varvec{C}}\right]$		
	Garment 1	Garment 2	Rubber	Garment 1	Garment 2	Rubber
${{\varvec{e}}}_{1}$	$2.94\pm 0.06$	$5.84\pm 0.14$	$5.52\pm 0.09$	$9.16\pm 0.81$	$41.18\pm 3.87$	$39.02\pm 3.15$
${{\varvec{e}}}_{2}$	$3.05\pm 0.09$	$3.75\pm 0.05$	$4.86\pm 0.36$	$16.48\pm 1.30$	$21.34\pm 2.81$	$37.67\pm 4.74$
${{\varvec{e}}}_{3}$	$3.71\pm 0.05$	$2.30\pm 0.10$	$6.88\pm 0.15$	$16.35\pm 0.91$	$1.50\pm 3.80$	$56.25\pm 2.95$
${{\varvec{e}}}_{4}$	$3.10\pm 0.05$	$2.41\pm 0.07$	$8.70\pm 0.10$	$12.08\pm 0.92$	$6.86\pm 3.13$	$71.90\pm 3.00$
${{\varvec{e}}}_{5}$	$4.21\pm 0.15$	$1.82\pm 0.04$	$7.17\pm 0.19$	$26.26\pm 1.71$	$4.63\pm 3.06$	$61.23\pm 3.01$
${{\varvec{e}}}_{6}$	$4.82\pm 0.07$	$2.55\pm 0.05$	$8.40\pm 0.10$	$26.71\pm 1.21$	$4.57\pm 3.23$	$73.00\pm 3.20$

## Discussion

### Prior work

Previous studies on textile electrodes intended for electrostimulation has investigated different parameters. Some have focused mainly on the manufacturing techniques of the electrodes and report material properties of the candidate electrodes^14,16^. Others have investigated comfort and user effects of a single electrode design^12,13^. Yet others have investigated how different design parameters influence the performance^27^. Only in one of the papers studying textile electrodes was reported that the actual time domain signals were used as discriminator when selecting a specific electrode design^17^. In this work we evaluated two types of bindings done with the same yarn and one ready-made fabric and compared them to the currently used conductive silicone rubber electrode. We chose three parameters for the comparison: the rise-time of the voltage, the total injected charge and the low frequency impedance magnitude. The over-arching idea of these three parameters was fidelity. Hence when designing the textile electrodes if the waveforms are equivalent to the once produced when using the rubber electrodes then that indicates that the textile electrodes perform in an equivalent way. The Mollii suit is a CE mark medical device that can be prescribed by physicians in Sweden and as such it fulfils with its intended use, and it is completely outside this work to investigate any aspect related to its performance.

### Evaluation of candidate electrodes

For the three textile candidate electrodes one can see the following features. Sample type B exhibit rise times twice as large compared to the benchmark, it has a higher net injected charge and a very low impedance, the friction is in parity with the benchmark. Sample type C have values closest to the benchmark for all three electrical parameters, the static friction is the lowest of all three textrode sample types namely 85% of the benchmark. The rise time is higher by 43%, the net injected charge is 96% of the benchmark and the low frequency impedance is 67%. Sample type D has a rise time smaller by 44%, net injected charge of 77% and a very small impedance, the friction is 89% of the benchmark. Overall, out of the textile samples, Type D has the most beneficial combined features. Hence that type was chosen as electrodes when constructing the garments.

#### Energy transfer to the body during stimulation cycle

The energy delivered to the body will be

9 $$E=\int Pdt=\int UI dt=\int Z {I}^{2}dt= Z\int {I}^{2}dt$$

Hence it is proportional to the integral of the square of the current. But as seen in the equation the impedance must be considered since that is where any energy can be dissipated. Now, if the current is measured at the return-path to the source the impedance includes two contacts together with the body impedance, thus we cannot from that kind of measurement alone claim any knowledge of how much that is delivered to the body. What can be said is that: if the contact impedance is small (at least the resistive or dissipative part) then most of the dissipated energy should be delivered to the body. Hence, a large contact impedance together with a large value of the integral will not necessarily mean a good performance of energy transmission, on the other hand a small contact impedance together with a large value of the integral will indicate a good performance. Of the two constructions made in house, the one that matches these criteria best is Type D. Type B has even better values in this sense. The experiment was not designed to investigate, in a systematic way, the impact the smoothness of the textrode has on the performance but the results seem to indicate that a smother surface is beneficial.

#### Skin-contact impedance

As pointed out in^1^ in general TES, a high resistivity electrode might be beneficial from a stimulation perspective. This is also what already van Boxtel found in 1977, but he also points out this will only be the case with current controlled TENS devices^28^. Also stated in those articles: if a high resistive electrode is chosen, one needs make sure that there are no inhomogeneities since they can cause very high current densities. When making textile electrodes, inhomogeneities are unavoidable and hence one cannot strive for a high resistive electrode in this case. This is one reason for choosing Type D over C. The variability of the impedance, especially at lower frequencies, is a well-known attribute of skin-electrode impedances^29^. Even more so in the case of dry electrodes. Also, the necessity to abandon a pure capacitor in a lumped element model in favour for a constant phase element is a widely used approach. Using a CPE yet another parameter for fitting is introduced. There is still an ongoing debate on how to explain the presence of such fractional capacitors, not only in skin–electrode contacts but in interfacial electro-chemistry in general. For our purposes, it is not so important at this stage to understand what physical situation they represent, but rather the functional behaviour they introduce. From the results for the patches, it is clear that regardless of whether a pure capacitor or a CPE is used, there is a roll-off in the impedance spectrograms for the skin–electrode contacts. This is true for all samples. All the samples display a behaviour that resembles a high pass filter, this is expected and in line with how skin-electrodes usually behave. Zhou et al. showed similar spectrograms in their experiments. What is also interesting in their results is the behaviour of the electrodes when interfaced with a metal plate instead of the human skin. It is clear from their measurements that the conventional hydrogel electrode interfacing a metal surface behaves as a parallel ${\varvec{R}}\left|\boldsymbol{ }\right|\boldsymbol{ }{\varvec{C}}$ link contrary to the textile electrodes who behaves as pure resistors. This shows that the reactive features of the skin–electrode impedances mainly stem from the interface, not from the electrode material^16^. To evaluate the candidate electrodes, we chose to aggregate four parameters in spider-charts, the candidate that most resembled the benchmark was chosen for the manufacturing of the garments. When it comes to the rise-time, an equal or lower value compared to the benchmark would be beneficial, the same goes for the low frequency impedance value and the static coefficient of friction. For the injected net charge, an equal or higher value compared to the benchmark was sought for. The only candidate that this is true for is the sample type D. Our original idea was that the binding in sample type C would introduce a higher effective contacting area thus lowering the resistive part of the impedance, it turned out that this was not the case. In fact, the amount of conductive yarn per unit area of type D is higher compared with type C. The knitting machines simulation program gives estimates that Type D uses ca 3.26% more yarn per unit area compared with Type C. This might explain the more favourable behaviour of type D. Another reason might be that part of the conductive material in the waves of type C is not being used effectively to contribute to the contacting surface. Instead leaving a space between the skin and the connection to the rest of the circuit. When the samples are pressed against the skin the intention was that the better part of the deformed waves would wrap around the skin, instead part of the waves are squeezed in directions parallel to the skin.

### Manufacturing and performance of the garments

The construction of the Mollii suit requires many production steps to integrate the electrodes and the conductive leads. The method we have used circumvents many steps in the production and further it minimisers the amount of residues in the production step. The Mollii suit is equipped with a multitude of zippers on the arms and legs. This is for ease of fitting the garment on the patient. The rubber electrodes present a high friction against the human skin, this is not the case with the textile electrodes. With the use of stretchable knitwear both a tight fit and ease of fitting is achieved. In addition, even more manufacturing steps are removed making the production faster and cheaper. Moineau et al. have recently demonstrated the production of similar kind of garments using circular knitting technique^2^. They also avoid the cut-and-sew step while positioning the electrodes. It is not clear from their text whether that technique also is able to seamlessly integrate the leads between the electrodes and the stimulation device. The flat-knitting intarsia-technique used in our study does this. Li et al. used flat knitting intarsia technique similar to the one used in this study, but they did not report a seamless integration of conductive leads and electrodes, instead they utilised the intarsia technique to achieve the pathways between the electrodes and the stimulation device. The electrodes were attached to the leads by means of metallic snap buttons^17^. The electrodes of the garments in this study were done using the binding of type D as explained above. The garments were designed to have a tight fit in order to secure good contact with the body. Thus, the area of the electrodes in the mechanically relaxed state was set to 6.5 × 5 cm^2, smaller than the 8 × 8 cm^2 of the patches. When worn by the test subject the area was stretched to ca 5.7 × 5.7 cm^2. This is still less than the 8 × 8 cm^2, so one would expect the magnitude of the low frequency impedances to be approximately twice the one measured on the patches. If the CPE is replaced by an ideal capacitor, then the expression of the impedance is

10 $${\varvec{Z}}=\frac{{{\varvec{R}}}_{{\varvec{p}}}}{1+{\left({\varvec{\omega}}{{\varvec{R}}}_{{\varvec{p}}}{\varvec{C}}\right)}^{2}}+{{\varvec{R}}}_{{\varvec{s}}}-{\varvec{j}}\frac{{\varvec{\omega}}{{\varvec{R}}}_{{\varvec{p}}}^{2}{\varvec{C}}}{1+{\left({\varvec{\omega}}{{\varvec{R}}}_{{\varvec{p}}}{\varvec{C}}\right)}^{2}}$$

If further we assume that ${{\varvec{R}}}_{{\varvec{p}}}$ and ${\varvec{C}}$ can be expressed by the ideal constitutive equations:

11 $${\varvec{R}}={\varvec{\rho}}\frac{{\varvec{L}}}{{\varvec{A}}},\boldsymbol{ }\boldsymbol{ }\boldsymbol{ }{\varvec{C}}={\varvec{\varepsilon}}\frac{{\varvec{A}}}{{\varvec{d}}}$$

Then

12 $${\varvec{Z}}=\frac{{\varvec{\rho}}\boldsymbol{ }{\varvec{L}}}{{\varvec{A}}\left(1+{\left(\frac{{\varvec{\omega}}{\varvec{\varepsilon}}{\varvec{\rho}}{\varvec{L}}}{{\varvec{d}}}\right)\boldsymbol{ }}^{2}\right)}+{{\varvec{R}}}_{{\varvec{s}}}-{\varvec{j}}\frac{{\varvec{\omega}}{\left({\varvec{\rho}}{\varvec{L}}\right)}^{2}{\varvec{\varepsilon}}}{{\varvec{d}}\boldsymbol{ }{\varvec{A}}\boldsymbol{ }\left(1+{\left(\frac{{\varvec{\omega}}{\varvec{\varepsilon}}{\varvec{\rho}}{\varvec{L}}}{{\varvec{d}}}\right)\boldsymbol{ }}^{2}\right)}$$

And as ${\varvec{\omega}}\to 0$ the resistive part is inversely proportional to the area. Predictions about the risetime is harder to make since it will depend to a higher degree also on ${{\varvec{R}}}_{{\varvec{s}}}$ and it is evident that the measured values can only in a very crude manner be approximated by the ideal situation. The garments were very easy to put on and the electrodes did not present any sharp edges, or high friction to the skin so from a wearer comfortability point of view this way of producing garments for electrostimulation is very attractive. From a producer point of view, it is also attractive in the sense that the knitting machine produces fabrics where the electrically active elements are already integrated hence one needs fewer production steps. The patterns can relatively easy be changed to fit different body types and to alter the sites of the electrodes which would make customisation feasible.

## Conclusions

The rubber electrodes present a rather high friction against the skin, thus making it cumbersome to put the garment on, especially for patient suffering from spasticity. The reason for this investigation was to determine if a textile electrode could be used for electrostimulation as a replacement of currently used conductive rubber electrodes and if a garment with electrodes, conductive leads between the electrodes and an electrostimulation-device could be produced in a way that only requires one single step of sewing. We have shown that this was achieved. The performance of the wholly textile system is not only satisfactory in that it delivers electrical energy impulse to the body as targeted but even beneficial when it comes to manufacturing and comfort for the wearer.

## Data Availability

The data sets and supplementary materials supporting the conclusions of this study are available upon request from the principal investigator [Fernando.Seoane@hb.se]. The authors commit to providing access to the data and materials promptly to researchers with a qualified purpose in accordance with the ethical approval vetting the collection of the data.
